# An Assessment of the Dietary Habits of Individuals with Migraine Living in Spain: An Exploratory Observational Cross-Sectional Pilot Study

**DOI:** 10.3390/nu17040686

**Published:** 2025-02-14

**Authors:** Vanessa Esteves-Mesquita, Álvaro Fernández-Cardero, Beatriz Sarriá, Izaskun Martín-Cabrejas

**Affiliations:** 1 School of Medicine, Complutense University of Madrid, Ciudad Universitaria s/n, 28040 Madrid, Spain; vaeste01@ucm.es; 2 Department of Metabolism and Nutrition, Institute of Food Science, Technology and Nutrition (ICTAN), Spanish National Research Council (CSIC), C/Jose Antonio Novais 6, 28040 Madrid, Spain; 3 Department of Nutrition and Food Science I, School of Pharmacy, Complutense University of Madrid, Ciudad Universitaria s/n, 28040 Madrid, Spain; alvafe22@ucm.es (Á.F.-C.);; 4 Department Food Technology, Veterinary Faculty, Complutense University of Madrid, 28040 Madrid, Spain; izaskmar@ucm.es

**Keywords:** migraine, chronic migraine, episodic migraine, headache, migraine-related disability, precipitating factors, nutrition, eating habits, Spain

## Abstract

Background/objectives: Eating habits have been proposed as a potential therapeutic approach for migraines; nevertheless, scientific evidence to support firm recommendations is lacking. Specifically, dietary habits in migraineurs living in Spain have not been investigated. Therefore, this study aimed to evaluate their dietary patterns and examine how these habits vary based on the frequency of migraine attacks or the degree of migraine-related disability. Methods: An exploratory, observational, cross-sectional pilot study was conducted on 260 individuals (18–64 years old) diagnosed with migraine in Spain. Data on diet, lifestyle, and migraine characteristics were collected with an online questionnaire consisting of a food frequency questionnaire and enquires about perceptions about diet, lifestyle, and different aspects related to migraines. Statistical differences were analyzed with the Kruskal–Wallis test, followed by Dunn’s post-hoc test, using JASP. Results: The consumption of plant-based foods was below the AESAN recommendations. No differences were observed in terms of food servings consumption across different migraine attack frequencies or levels of migraine-related disability. Both the chronic migraine group and the severe disability group showed differences in the consumption of some foods considered as migraine triggers (such as chocolate, cured cheese, cured meats, and alcoholic beverages). Moreover, people who suffered from infrequent migraine consumed significantly more caffeine than those who had chronic migraine. Conclusion: It remains unclear whether avoiding dietary migraine triggers is driven by the biological effects of certain food compounds or influenced by dietary perceptions and unfounded beliefs. Thus, further research on the role of diet in migraine management is necessary.

## 1. Introduction

Migraine, a prevalent and complex neurological disorder, is influenced by both genetic and environmental factors and is characterized by debilitating headaches along with transient motor and somatosensory disturbances such as photophobia, phonophobia, osmophobia, nausea, and vomiting [1,2]. Migraine is classified as episodic when patients experience headaches on fewer than 15 days per month and chronic when they persist for 15 or more days each month. The number of days that the patient suffers from headaches influences migraine-related disability and represents a key factor to monitor the progression of the condition from episodic to chronic [3].

This pathology exhibits a high global prevalence, with roughly one-third of adults aged 18 to 65 diagnosed with the disorder, according to data from the World Health Organization (WHO) [4,5]. In Spain, the Spanish Society of Neurology (SEN) estimates that migraine affects approximately 5 million people. Among these, 70% experience severe disability, and 14% suffer moderate disability [6]. Of the diagnosed cases, 1.5 million individuals endure chronic migraines, with impaired quality of life and reduced productivity, resulting in a sixfold increase in disability compared to episodic migraines [6]. In fact, according to the 2019 Global Burden of Disease (GBD) study, migraine has been identified as the second leading cause of disability among the adult population and the foremost cause in women, which aligns with its threefold higher prevalence in women compared to men [7,8].

Understanding the factors contributing to migraines can help individuals identify their personal triggers and manage migraine attacks more effectively. In recent years, there have been concerted efforts to identify effective strategies for the prevention and treatment of neurological disorders, outstanding strategies that involve dietary interventions. Emerging research on general dietary patterns suggests that the eating habits of migraine patients may have an impact on disease burden and quality of life [2,7]. Adopting a healthy diet with adequate nutrients intake can positively impact neuroreceptors, cerebral glucose metabolism, the sympathetic nervous system, and neuroinflammation [9]. However, to date, there is limited evidence to suggest that certain dietary patterns are associated with a reduction in migraine episodes [10]. A few studies have indicated that a balanced diet rich in whole grains, legumes, fruits, and vegetables may offer neuroprotective and anti-inflammatory effects against migraine disease due to their high content of phytochemicals, fiber, and micronutrients [11,12].

These patterns agree with the recently released Spanish Agency for Food Safety and Nutrition (AESAN) dietary guidelines (2022) which emphasize the importance of increasing the consumption of nutrient-dense, plant-based foods while reducing the consumption of animal-based foods to prevent and manage chronic diseases [13]. Other countries have adopted similar recommendations, such as the recent Scientific Report of the 2025 Dietary Guidelines Advisory Committee, reflecting a global trend towards shifting dietary patterns to a more plant-based approach [14]. However, it should not be disregarded that migraine may compromise the adoption of a healthy diet, because some patients may have altered neurotransmitters, hormones, and adipokines levels, affecting food choices and metabolism [15].

Currently, some dietary strategies such as elimination diets (targeting potential dietary triggers) are being considered as complementary interventions to mitigate the impact of this disease. Although the effects of dietary triggers can vary between individuals, certain food components can be commonly associated with migraines, such as biogenic amines in aged cheeses, cured meat, fermented foods, pickled foods, and alcoholic beverages [16,17,18]; monosodium glutamate contained in processed foods (soups, chips, and frozen meals) and snacks [19]; aspartame in sugar-free soda and gums as well as low-calorie or diet foods [20]; nitrates and nitrites in processed, deli, and canned meats [21]; chocolate and hot chocolate drinks due to the content in caffeine and phenylethylamine [22]; alcoholic beverages [17]; citrus fruits [23]; and highly processed or refined foods, especially those high in sugar, sodium, or artificial additives [23]. Caffeine could be included in this list, although it may also have the opposite effect by relieving migraine symptoms due to its analgesic properties. Alternatively, migraines may be triggered by the withdrawal from excessive caffeine consumption [24].

As far as the authors are aware, no previous work has studied dietary habits or other related lifestyle factors in a Spanish population who suffer from migraines. Therefore, this pilot study aims to analyze the dietary habits of migraine patients in Spain, focusing on differences based on attack frequency and migraine-related disability level. Additionally, it explores dietary triggers and food-related perceptions and beliefs that may influence their dietary choices.

## 2. Materials and Methods

### 2.1. Study Design

An exploratory observational cross-sectional pilot study was conducted in men and women between 18 and 64 years old, diagnosed with migraine and living in Spain. Participants in the study were recruited through the Spanish Association of People with Migraine (AEMICE), using their newsletter. Additionally, the survey was disseminated through social media platforms, including Facebook, Instagram, and Twitter/X and among relatives, co-workers, and acquaintances of both the research team and the participants. To ensure the accuracy and reliability of the respondents, the survey included a question regarding the medical diagnosis of migraine, along with eight specific questions related to migraines used to ensure the consistency of the responses. This study received approval from the Ethics and Biosafety Committee for Research of the Complutense University of Madrid, under protocol number CE_20230209-15_SAL, on 30 March 2023. Furthermore, the ethical recommendations of the Declaration of Helsinki were followed. Personal data were anonymous for subsequent analysis and handled according to the EU General Data Protection Regulation (Regulation EU 2016/679 of the European Parliament and of the Council, 27 April 2016) and Spain’s Organic Law 3/2018 on Personal Data Protection and Guarantee of Digital Rights. Furthermore, the study protocol followed the ethical recommendations of the Declaration of Helsinki. The inclusion criteria for this study were as follows: participants must have a diagnosis of migraine according to established medical diagnosis criteria, be between 18 and 64 years old, reside within Spanish territories, and have access to the internet.

### 2.2. Questionnaire Design

Participants completed a survey designed for the present study via an anonymous online questionnaire hosted on the Google Forms platform. The survey consisted of 39 questions divided into eight sections addressing demographic data (place of residence), basic anthropometric information (weight and height), and pathological and pharmacological history. The frequency of migraine attacks per month was used to classify the type of migraine, in accordance with the criteria outlined in the Manual de Práctica Clínica en Cefaleas—Recomendaciones Diagnóstico-Terapéuticas, published by the Spanish Society of Neurology (2020). Thus, migraine was categorized as episodic (<15 days/month), which was further subdivided into infrequent episodic (1–9 days/month) and frequent episodic (10–14 days/month), or chronic (≥15 days/month) [6]. In addition, they completed the following questionnaires: Migraine Disability Assessment Scale (MIDAS) Score, Visual Analog Scale (VAS), physical activity based on the World Health Organization’s Global Recommendations on Physical Activity for Health, sleep hygiene (hours of sleep), and consumption of toxic substances (tobacco, alcohol). The MIDAS Score classifies individuals based on functionality into the following categories: no disability (0–5 days), mild disability (6–10 days), moderate disability (11–20 days), and severe disability (>21 days) [25]. Meanwhile, the VAS categorizes pain severity into three groups: mild pain (1–3 points), moderate pain (4–7 points), and severe pain (8–10 points) [26]. The participants’ physical activity was categorized based on the WHO’s recommendations into the following groups: those who do not engage in any form of physical activity, those who engage in less than 150 min of moderate aerobic activity or less than 75 min of vigorous activity per week, those who engage in at least 150 min of moderate aerobic activity or 75 min of vigorous activity per week, those who engage in at least 300 min of moderate aerobic activity or 150 min of vigorous activity per week, and finally, those who engage in more than 300 min of moderate aerobic activity or more than 150 min of vigorous activity per week [27].

For dietary monitoring, a food frequency questionnaire (FFQ) adapted from the PREDIMED study was employed to calculate the daily and weekly portions consumed from different food groups (cereals, tubers, legumes, nuts, vegetables, fruits, meats, fish, eggs, and dairy products) [28]. In addition to the FFQ, the Dietary Diversity Score (DDS) was incorporated to assess the diversity within food groups based on a healthy and balanced diet. Participants are asked whether they consumed at least one food item from specific groups in the past three days, with responses Scored as “Yes” (1 point) or “No” (0 points). The DDS evaluates variety within five main groups: grains and cereals, vegetables, fruits, dairy products, and meats and meat substitutes [29]. This study focused on analyzing 30 out of the 39 questions included in the survey. The survey was conducted between March and May 2023.

### 2.3. Diet Assessment

The total number of servings of fruits, vegetables (separately and combined), dairy products, cereals (total, refined, and whole grains), nuts, legumes, oils, tubers, eggs, meat, and fish were calculated. The serving size in grams was specified next to each food item in the FFQ.

Additionally, daily consumption of caffeine-rich foods and drinks, including coffee, tea, cola-based caffeinated drinks, chocolate and cocoa powder, alcoholic drinks and foods rich in biogenic amines, nitrites and nitrates, such as semi-cured and cured cheeses, cured meats, cooked ham, and pickled foods, was also calculated. Portion sizes for these foods and dinks were estimated using data from the food frequency questionnaire, which served as the basis for the survey. Moreover, the caffeine content of various drinks was estimated attending to the scientific opinion on caffeine safety published by the European Food Safety Authority (EFSA) in 2015 [30]. Based on this scientific opinion, the caffeine content was estimated to be approximately 80 mg per cup of coffee (60 mL), 50 mg per cup of tea (220 mL), 40 mg per standard can of cola (355 mL), 25 mg per serving of cocoa powder (50 g) and 10 mg per chocolate milk bar (50 g) [30].

The main results of the article detail the number of servings for both consumers and non-consumers. Additionally, the Supplementary Materials section includes the calculations of servings, excluding non-consumers from each food group or item.

### 2.4. Sample Saze Calculation

The sample size calculation for our study was based on the difference in fruit consumption observed in the study “Comparison of Diet Quality Between Women With Chronic and Episodic Migraine” by Hajjarzadeh et al. [31] aimed at comparing the diet quality between women with chronic and episodic migraine. Fruit consumption was chosen as the primary variable because it has been associated with reduced migraine severity, making it a relevant factor to investigate in this context [31,32,33,34,35,36]. The required sample size was calculated with the Software Sample Size Calculator (version 1.062) (Robin Ristl, University of Vienna) [37], considering that the mean value for fruit consumption based on the Healthy Eating Index-2015 for people with sporadic migraine (group 1) was 4.35 (SD = 0.84), while for people with chronic migraine (group 2) it was 3.87 (SD = 1.19), yielding a mean difference in −0.48. A power of 80% (β = 0.20) was set to ensure sufficient sensitivity. The confidence level was set at 95% for a type I error (two-tailed Zα-Score: 1.96). Afterwards, the significance level was adjusted using the Bonferroni correction, as our analysis involves up to six comparisons based on the MIDAS Score classification scale, which includes four classification groups (no disability, mild disability, moderate disability, and severe disability). This correction was applied to reduce the risk of type I errors, ensuring the reliability of our findings. Based on these parameters, the required sample size was determined to be 254 participants, similar to Hajjarzadeh et al. [31] which was 280.

### 2.5. Statistical Analysis

For this observational, cross-sectional pilot study a convenience sample was used, consisting of participants diagnosed with migraines who reside in Spain. Data were analyzed using JASP (version 0.18.3.0). Categorical variables were expressed as the absolute frequencies (*n*) and relative frequencies in percentage (%). The chi-squared test was used for comparisons between groups on categorical variables. The Kolmogorov–Smirnov test or the Shapiro–Wilk test was applied to assess the assumption of normality in quantitative variables depending on the sample size (Kolmogorov–Smirnov test for groups *n* > 50 or Shapiro–Wilk for groups *n* < 50). Moreover, quantile–quantile plots (Q–Q plots) and Boxplots were used to corroborate normality and to identify extreme values. If variables were normal, data were expressed as mean ± standard deviation, while non-normally distributed variables were expressed with median and the 25th percentile and the 75th percentile in parentheses (the interquartile range [IQR]). For quantitative variables that followed a normal distribution, the one-way analysis of variance (ANOVA) was used, while for non-normally distributed variables, the Kruskal–Wallis H test was performed.

As food groups and specific food items (caffeine-rich foods and drinks, alcoholic beverages, foods rich in biogenic amines, nitrites and nitrates, and daily caffeine intake) did not follow a normal distribution, the non-parametric Kruskal–Wallis H test was performed for the primary objective of this study. This analysis was conducted across groups categorized by different migraine frequencies (infrequent, frequent, or chronic) and disease-related disability levels determined by the MIDAS Score. Post-hoc pairwise comparisons were carried out employing Dunn’s test, with Bonferroni correction and *p*-values under 0.05 were considered significant.

## 3. Results

**Figure 1:**
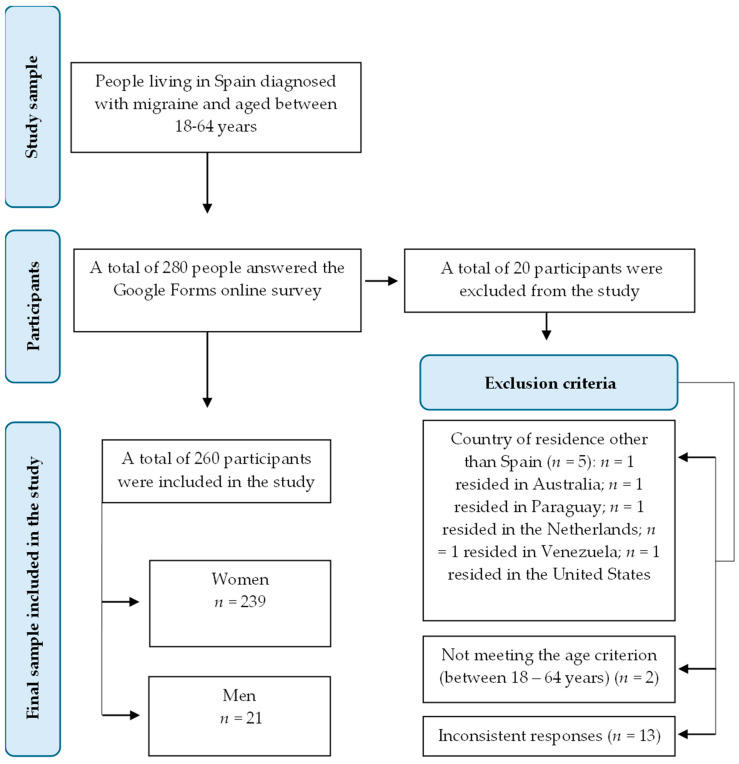
Flowchart of response selection for the development of the observational study.

The initial study sample consisted of 280 participants. At the end, the final sample included in this study was formed by 260 subjects, as those who did not meet the inclusion criteria were excluded (Figure 1).

### 3.1. Descriptive Data of the Study

Table 1 shows the clinical characteristics of the study population according to the frequency of migraine attacks, type of migraine, disease-related disability levels, and disease-related pain levels determined by the MIDAS Score and the VAS, respectively (Table 1).

Demographic and lifestyle characteristics of the subjects who participated in the present study are displayed in Table 2 (Table 2).

### 3.2. Data on the Dietary Consumption of Participants

Table 3 presents the median values and the 25th percentile and the 75th percentile for the consumption of various food groups and drinks by participants, alongside dietary recommendations provided by AESAN (Table 3). Supplementary Materials presents data on food group consumption, excluding individuals who did not consume any items from those groups. The consumption of sunflower oil, high-oleic sunflower oil, and coconut oil was very low among the studied population, as shown in the Supplementary Materials (Table S1). Consequently, no data on these oils were included in Table 3.

**Figure 2:**
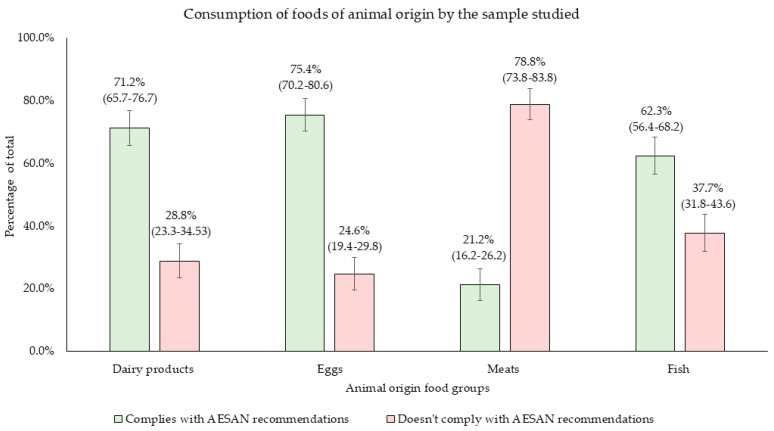
Consumption of animal-based foods among participants. Means are presented with 95% confidence intervals (CI).

Figure 2 illustrates the average consumption of portions from different animal-based food groups compared to AESAN guidelines. The results show that 71.2% of participants adhered to recommendations for dairy intake (≤3 servings/day), 62.3% met recommendations for fish (≥3 servings/week), and 75.4% complied with egg intake guidelines (≤4 servings/week). However, 78.8% exceeded the recommended weekly intake of meat (≤3 servings/week) (Figure 2).

**Figure 3:**
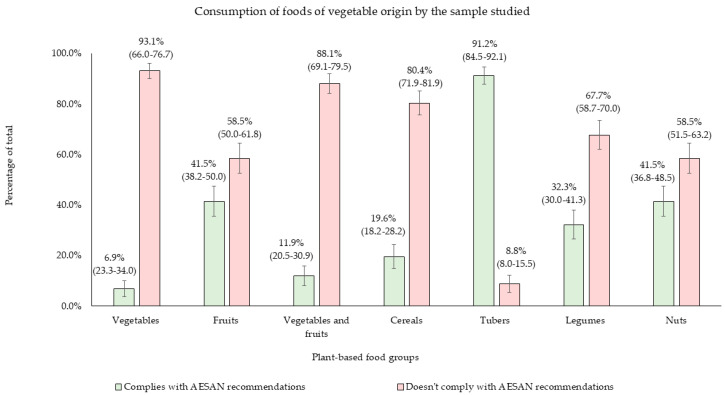
Consumption of plant-based foods among participants. Means are presented with 95% confidence intervals (CI).

Figure 3 depicts the average consumption of portions from various plant-based food groups compared to AESAN recommendations. The findings reveal that 93.1% of respondents did not meet the daily recommended intake of vegetables (≥3 servings/day), 58.5% fell short of fruit recommendations (≥2 servings/day), and 88.1% failed to reach the combined fruit and vegetable intake target (≥5 servings/day). The consumption of legumes (≥4 servings/week), nuts (≥3 servings/week), and cereals (3–6 servings/day) were also below recommendations. The only plant-based food group meeting the guidelines was tubers, with 91.2% of participants consuming one or fewer servings per day (Figure 3).

**Figure 4:**
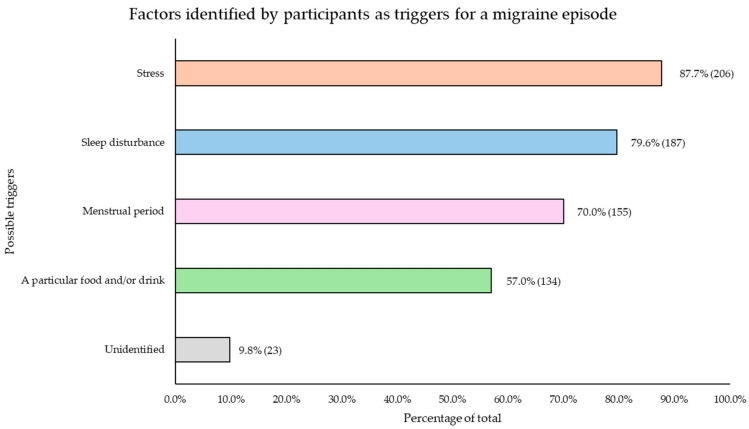
Factors identified by participants as triggers for a migraine episode. Data are presented as the relative frequency (%) and absolute frequency (n).

When participants were queried regarding their self-perceived susceptibility to migraine episodes under specific circumstances, a total of 90.4% (*n* = 235) of the respondents reported having identified one or more situations that predisposed them to migraine attacks. The most cited triggers were experiencing some form of stress (*n* = 206), disruptions to sleep patterns—whether due to insufficient sleep duration or lack of restorative sleep (*n* = 187)—and, in the case of women, the onset of the menstrual period (*n* = 155). Additionally, the consumption of specific foods and/or drinks was also highlighted as a self-perceived contributing factor (*n* = 134) (Figure 4).

**Figure 5:**
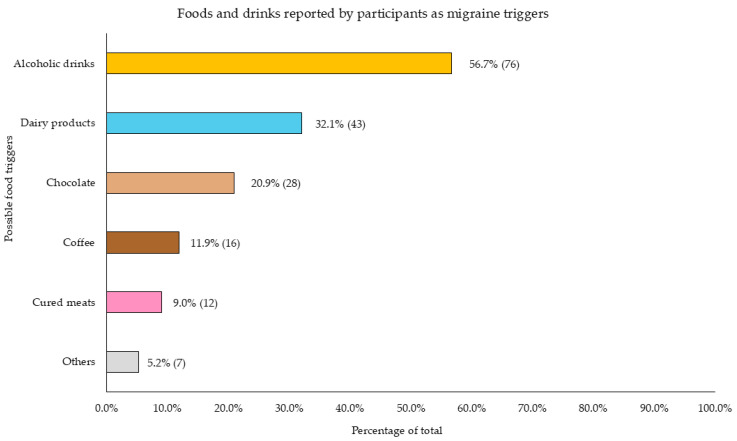
Foods and drinks identified by participants as being associated with the onset of migraine attacks. Data are presented as the relative frequency (%) and absolute frequency (n).

Among those participants who identified food and/or drinks as possible migraine triggers, alcoholic drinks were the most frequently reported dietary factor, cited by 56.7% of respondents. Dairy products were the second most mentioned trigger, identified by 32.1% of participants, followed by chocolate (20.9%), coffee (11.9%), and cured meat (9%). A further 5.2% of participants mentioned various other less frequently reported triggers, including onions, citrus fruits, and tea (Figure 5).

No significant differences were identified between the groups established according to frequency of migraine attacks when comparing the number of servings of different food groups (*p* > 0.05) (Table 4). The consumption of specific foods and drinks identified by participants as potential migraine triggers based on their personal beliefs (Figure 5) was generally low. This was particularly evident for soft cola drinks, cocoa powder, alcoholic drinks, and semi-cured and cured cheeses, as highlighted in the Supplementary Materials (Table S2). The percentage of non-consumers was considerable for these items, with 78.8% for soft cola drinks, 73.8% for cocoa powder, 74.6% for alcoholic beverages, and 66.9% and 70.8% for semi-cured and cured cheeses, respectively (Table S2). In contrast, significant differences were observed concerning the consumption of chocolate (*p* = 0.032), total caffeine (*p* = 0.046), alcoholic drinks (*p* = 0.016), and semi-cured cheeses (*p* = 0.008). Total caffeine consumption by migraine type and source is presented in Figure S1 in the Supplementary Materials section. According to post hoc pairwise comparisons conducted using Dunn’s test with Bonferroni adjustment, individuals with chronic migraine consumed statistically less chocolate compared to those with frequent migraine (*p* = 0.044). Additionally, daily consumption of caffeine and alcoholic beverages was significantly lower among individuals with chronic migraine compared to those with infrequent migraine (*p* = 0.044 and *p* = 0.016, respectively). Similarly, the daily intake of semi-cured cheeses was found to be lower in individuals with chronic migraine compared to both those with frequent migraine (*p* = 0.049) and those with infrequent migraine (*p* = 0.010) (Table 5).

No statistical differences were identified between the groups concerning disease-related disability according to the MIDAS Score, except for daily consumption of virgin olive oil (*p* = 0.041; Table 4), although no intergroup differences were obtained (*p* > 0.05). Conversely, significant differences were observed between groups with different levels of migraine-related disability when analyzing the consumption of alcoholic drinks (*p* = 0.042), cured cheeses (*p* = 0.008), cured meats (*p* = 0.020), and cooked ham (*p* = 0.046), which are commonly reported as potential triggers of migraine episodes. A post hoc pairwise comparison was subsequently conducted showing that individuals without disability consumed significantly larger quantities of alcoholic drinks daily compared to individuals with severe disability (*p* = 0.032; Table 5). Regarding the consumption of cured cheeses, individuals experiencing moderate disability consumed higher amounts than those with severe disability (*p* = 0.018). In contrast, for cured meats and cooked ham, post hoc analysis revealed no significant differences between the groups (*p* > 0.05; Table 5).

Furthermore, the median DDS for the entire sample was 10.0 [8.0–11.0]. In addition, a comparison of the total DDS in relation to migraine attack frequency and disease-related disability according to the MIDAS Score has been included in the Supplementary Materials section (Table S6). No significant differences were observed between the different migraine attack frequency groups or between the various disability groups based on the MIDAS Score (Table S6).

## 4. Discussion

The novelty of this work consists of the analysis of dietary habits and other related lifestyle factors in a Spanish population who suffer from migraines. In the present cross-sectional study, the participants were predominantly females (91.9%) and exhibited a daily smoking prevalence of 15%, consistent with the 16.4% reported for women in Spain by the National Institute of Statistics (INE) [38]. Previous studies have identified cigarette smoking as a precipitating factor for migraine attacks [39]. In contrast, alcohol abstinence was reported by 90.7% of participants, a markedly higher proportion compared to the 43.2% of women and 25.4% of men in Spain who abstain from alcohol, according to INE data [38]. This divergence is likely attributable to the specific characteristics of our cohort, which consists of migraine patients who frequently identify alcohol, smoking, and other dietary factors as migraine triggers (Figure 5). Moreover, around 60% of the study population had a sedentary or low physical activity lifestyle, six out of ten individuals reported good sleep hygiene, and 14.2% of participants were polymedicated (Table 2). The most common type of migraine was episodic, reported by 71.5% of respondents. A significant proportion of participants experienced severe disability and pain associated with migraine attacks (73.8% and 71.9%, respectively) (Table 1).

Regarding hydration, the study population showed a low median daily water intake of 600 mL/day (Table 3). Emerging evidence highlights a significant inverse relationship between daily water intake and migraine severity, with increased hydration associated with reductions in migraine-related disability, pain intensity, headache frequency, and duration [40]. This relationship may be explained by the physiological role of hydration in migraine pathophysiology. Dehydration and insufficient fluid intake are known to reduce blood volume, impairing cerebral oxygen delivery and potentially triggering headaches. Adequate hydration stabilizes plasma osmolality and ion concentration, which may contribute to the alleviation of migraine symptoms [41]. However, stratification of participants by migraine attack frequency and migraine-related disability revealed no significant differences in beverage consumption patterns across groups (Table 4).

Focusing on the analysis of dietary habits, most individuals in the study group presented a high dietary diversity based on their DDS (Table S6), and the majority adhered to the AESAN dietary recommendations for the Spanish population regarding animal-based foods, except for meat consumption, which exceeded the recommended levels (Figure 2). For plant-based foods, daily intake of fruits, vegetables, and cereals, as well as weekly consumption of legumes and nuts, fell below the recommended thresholds (Figure 3). No significant differences were observed between the groups with different types of migraines (according to the frequency of attacks) or between groups of disease-related disability (according to the MIDAS Score) regarding food servings, indicating uniform consumption among participants (Table 4). Building on this, the present findings regarding the dietary consumption of the participants align with the 2019 European Union report, which indicated that only 19.3% of the Spanish population consumed five or more servings of fruits and vegetables per day—a figure comparable to the 11.9% observed in this study [42].

It is noteworthy that the present results are consistent with the ANIBES study, which was conducted on a representative sample of Spanish adults aged 18–75 years in 2016. The study indicates that approximately 84% of the population does not meet the recommended daily cereal intake [43]. Similarly, in the current migraine population, the prevalence of inadequate cereal intake was 80.4% (Figure 3). Again, comparing the two studies, there is also an agreement regarding olive oil consumption, since in ANIBES the average consumption was 11 L of olive oil annually, equating to roughly three daily servings, an amount similar to the 2.4 servings observed in this study [43]. However, weekly legume consumption differed, as in the present study, 3.9 servings/week were observed, in contrast to the average of 1 serving/week in ANIBES. This increase in legume consumption may be linked to the growing recognition for nutritional benefits, environmental sustainability, economic accessibility, and alignment with modern dietary trends [44]. As awareness of the environmental impact of food choices continues to grow, women may be more inclined to adopt plant-based diets and make legumes a staple component [45]. Consequently, the higher legume intake observed in this study could be partly explained by the predominance of women in the study population, as they often prioritize health due to a combination of physiological, psychological, and sociocultural factors [46].

Regarding animal-based food consumption, the ANIBES study explains that 66.4% of Spaniards consume three or fewer dairy servings daily, only 25% meet or exceed three weekly fish servings, and 59% consume more than one daily serving of meat [43]. Similar trends were observed in this study, with 71.2% consuming three or fewer dairy servings daily and 78.8% exceeding three weekly servings of meat (Figure 2). However, fish consumption was higher among the participants of the present study, with 62.3% consuming three or more weekly servings [43]. This discrepancy could be explained, as with legumes, by the fact that the sample is formed mainly by females [46]. Women tend to consume more fish than men due to a combination of health considerations, dietary preferences, sustainability concerns, cultural norms, and targeted marketing [46].

The reduction in the consumption of plant-based foods and the increase in the intake of animal-based foods can be attributed to a shift away from the Mediterranean diet—rich in fruits, vegetables, whole grains, legumes, and extra virgin olive oil—towards a Western diet characterized by a high consumption of animal-based foods, added sugars, and saturated fats [47,48]. Plant-based diets, rich in antioxidants and prebiotics, may help reduce migraine frequency and severity due to their anti-inflammatory properties [11,49]. Inflammation is a known contributor to headaches, and the beneficial components of plant-based foods can positively influence the gut–brain axis by promoting healthy gut microbiota, which has been linked to migraine triggers [50,51]. Recent studies highlight that dietary patterns significantly impact migraine symptoms, with plant-based diets—rich in fruits, vegetables, whole grains, and plant-based proteins—showing promise in reducing the frequency of migraine attacks. In contrast, diets high in processed foods, sugars, and alcohol are associated with more severe symptoms [11]. For instance, a large cross-sectional study found that higher fruit and vegetable consumption was associated with a lower likelihood of primary headaches [32]. Additionally, the DASH diet, which emphasizes plant-based foods, has been linked to reduced headache severity and duration in migraine patients [34]. While the exact mechanisms remain unclear, the growing body of evidence underscores the potential of plant-based diets as a therapeutic approach for migraine management [52].

Interestingly, participants in this study reported self-perceived migraine triggers, including stress, sleep disturbances, menstrual periods, and dietary factors (Figure 4). Specific foods identified as potential triggers were alcohol, dairy products, chocolate, coffee, and processed meats (Figure 5). A significant proportion of participants avoided these foods, consistent with evidence suggesting that individuals with migraines often eliminate perceived dietary triggers to reduce attack frequency or severity [53]. This behavior reflects the complex interplay of dietary, hormonal, and environmental factors in migraine triggers [53]. Migraine patients in this study consumed lower amounts of these foods compared to the general Spanish population, according to findings from another cohort of the ANIBES study [54]. In that study, the average daily consumption was 0.05 servings of processed meats, 0.37 servings of cheese, 0.20 servings of chocolate, 1.57 servings of coffee, and 0.76 servings of alcoholic beverages. These consumption levels were higher than those observed among individuals with migraines included in the present study (Table S5).

In relation to this avoidance behavior towards certain foods, the present study observed significantly lower consumption of chocolate in individuals with chronic migraine compared to those with frequent migraine (*p* = 0.032). Additionally, the daily intake of semi-cured cheeses was found to be lower in individuals with chronic migraine compared to both those with frequent migraine (*p* = 0.049) and those with infrequent migraine (*p* = 0.010) (Table 5). The potential migraine-triggering effects of semi-cured cheese may be attributed to its biogenic amine content, particularly tyramine, which has been implicated in altering neurotransmitter levels and vascular function [16,18,55]. Chocolate, on the other hand, contains methylxanthines such as theobromine and caffeine as well as biogenic amines like phenylethylamine, tyramine, and histamine compounds that may also influence neurovascular pathways and migraine pathophysiology [22,56].

Moreover, the total intake of caffeine was notably lower in individuals with chronic migraine compared to those with infrequent migraine (*p* = 0.044) (Table 5; Figure S1). The role of caffeine in migraines is complex and often contradictory. While caffeine has been reported to act as a trigger for some individuals, it may also provide relief in others when used judiciously. This dual effect could be related to caffeine’s influence on adenosine receptors, its ability to modulate cerebral blood flow, and its potential to contribute to withdrawal headaches when consumed habitually and then discontinued abruptly [57]. Additionally, caffeine has been studied for its potential analgesic effects. A study found a significant association between high caffeine consumption and the prevalence of infrequent headaches, suggesting that individuals suffering from chronic headaches tend to avoid caffeine consumption to prevent worsening their symptoms [57].

Alcoholic beverages were also identified as a significant migraine trigger, with 56.7% of participants attributing their migraine episodes to alcohol consumption (Figure 5), consistent with existing literature on the beliefs of individuals with this condition [53]. Reflecting this perception, a notably high proportion of participants (74.6%) reported abstaining from alcohol (Table S2). Individuals with chronic migraine and severe disability reported consuming considerably less alcohol daily compared to those with infrequent migraine and no disability (*p* = 0.016; *p* = 0.032, respectively) (Table 5). The effects of alcohol on migraines are complex and influenced by individual variability. Alcohol may act as a trigger through mechanisms like vasodilation, which can activate migraine pathways, and dehydration, a common trigger due to its diuretic effects [17]. Biogenic amines such as histamine and tyramine, present in certain drinks like red wine, can also disrupt vascular and neurotransmitter functions [58,59]. Additionally, alcohol may alter serotonin levels, interfere with sleep quality, and provoke delayed headaches during withdrawal [5,60].

To end, it is important to note that although the present work is focused on eating habits in Spanish subjects who suffer from migraines, this disorder is a complex neurological event that can be triggered by a variety of factors, including genetic, environmental, lifestyle, and physiological influences [5]. Thus, in future studies it will be necessary to analyze these factors in depth using a multidisciplinary approach.

### Limitations

The main limitation of this pilot study is that it is based on a non-representative sample which may affect the generalizability of the findings. In the present study, most participants reported episodic migraines and presented severe disability according to the MIDAS Score. Furthermore, the sample consisted of a significantly higher proportion of female participants, which is consistent with the higher prevalence in women compared to men. Notwithstanding, the sample size was sufficient to carry out the main objective of the study, providing a foundational basis for future research. Another potential limitation of this study is that the timing of food consumption was not included, as this is not captured by the food frequency questionnaire used. In addition, physical activity levels were self-reported based on WHO recommendations.

## 5. Conclusions

In this exploratory observational cross-sectional pilot study conducted among a convenience sample of a Spanish population diagnosed with migraines, plant-based food consumption was below AESAN recommendations, while meat consumption was excessive. However, no differences were found in food groups consumption when comparing people with different migraine attack frequency or migraine-related disability. 

Both the group with chronic migraines and the group with severe disability showed lower consumption of some foods considered as migraine triggers (such as chocolate, cured cheese, cured meats, and alcoholic beverages). Additionally, individuals with chronic migraines had lower caffeine intake compared to those with infrequent migraines. 

It remains unclear whether these differences are due to the biological effects of certain compounds in these migraine triggers (such as caffeine, alcohol, biogenic amines, and nitrates/nitrites) and/or stem from perceptions and unfounded beliefs. Therefore, the effect of these compounds should be studied in future research.

## Figures and Tables

**Table 1:** Migraine-related clinical characteristics of the sample population.

	Total *n* = 260
Frequency of migraine attacks (numer per month) *	8.0 [5.0–17.3]
	n = 256
Type of migraine (number of migraine attacks per month) *	8.0 [5.0–17.0]
Episodic, (<15 days/month) n (%)	183 (71.5%)
Infrequent episodic attacks (1–9 days/month), n (%)	134 (52.3%)
Frequent episodic attacks (10–14 days/month), n (%)	49 (19.2%)
Chronic, (≥15 days/month) n (%)	73 (28.5%)
MIDAS Score *	36.0 [20.0–76.5]
No disability, n (%)	16 (6.2%)
Mild disability, n (%)	21 (8.1%)
Moderate disability, n (%)	31 (11.9%)
Severe disability, n (%)	192 (73.8%)
VAS Scale *	8.0 [7.0–8.5]
Mild pain, n (%)	1 (0.4%)
Moderate pain, n (%)	72 (27.7%)
Severe pain, n (%)	187 (71.9%)

The superscript * denotes data that do not follow a normal distribution. Quantitative variables are presented as the median with the 25th percentile and the 75th percentile in parentheses depending on the normality of the data.

**Table 2:** Demographic and lifestyle characteristics of the total studied population.

	Total *n* = 260
Age (years) (Mean ± SD)	37.8 ± 10.3
Sex, n (%)	
Women	239 (91.9%)
Men	21 (8.1%)
BMI (kg/m^2^) (Median [25th percentile and the 75th percentile]) ^a^	23.6 [21.1–27.3]
Underweight, n (%)	16 (6.2%)
Healthy weight, n (%)	149 (57.3%)
Overweight, n (%)	54 (20.7%)
Obesity n (%)	41 (15.8%)
Alcohol, n (%)	
Yes	24 (9.2%)
No	236 (90.7%)
Smoke, n (%)	
Yes	9 (15.0%)
No	221 (85.0%)
Physical activity, n (%)	
Does not engage in any form of physical activity	62 (24.7%)
<150 min of moderate aerobic activity or <75 min of vigorous activity	83 (33.1%)
At least 150 min of moderate aerobic activity or 75 min of vigorous activity	57 (22.7%)
At least 300 min of moderate aerobic activity or 150 min of vigorous activity	24 (9.5%)
>300 min of moderate aerobic activity or >150 min of vigorous activity	25 (10.0%)
Hours of sleep per day, n (%)	
<7 h daily	91 (35.1%)
7–8 h daily	142 (54.8%)
≥8 or more hours daily	26 (10.0%)
Medications, n (%)	
<5 medications/day	223 (85.8%)
≥5 medications/day	37 (14.2%)

Quantitative variables are presented as mean ± standard deviation (SD) and as median with the 25th percentile and the 75th percentile in parentheses, depending on normality. The superscript “a” indicates data that does not follow a normal distribution. Categorical variables are expressed as absolute frequencies (n) and relative frequencies (%). BMI: body mass index.

**Table 3:** Average daily and weekly consumption frequency of different food groups in the total study population.

Food Groups	Frequency Food Comsumption	AESAN Recommendations [13]
Dairy products (milk, yogurt, cheese, etc.)	2.3 [1.0–3.3]	≤3 servings/day
Eggs *	3.6 [1.0–4.0]	≤4 servings/week
Meats (chicken, turkey, beef, pork, etc.) *	5.9 [3.0–7.0]	≤3 servings/week
Fish (hake, sole, salmon, tuna, seafood, etc.) *	4.8 [2.0–7.0]	≥3 servings/week
Vegetables (green beans, spinach, etc.)	1.0 [0.0–1.1]	≥3 servings/day
Fruits (citrus, apple, pear, etc.)	1.6 [0.5–2.1]	≥2 servings/day
Vegetables and fruits	2.6 [1.1–3.0]	≥5 servings/day
Tubers (potato, sweet potato, etc.)	0.5 [0.0–0.7]	≤1 servings/day
Legumes (lentils, beans, etc.) *	3.9 [1.0–5.0]	≥4 servings/week
Nuts (walnuts, cashews, etc.) *	4.7 [0.0–7.0]	≥3 servings/week
Cereals	2.1 [1.0–2.8]	3–6 servings/day
Refined cereals	1.0 [0.3–1.3]	≤50% of servings
Whole grains	0.7 [0.0–1.0]	≥50% of servings
Oil	2.3 [1.0–2.5]	Preferential consumption of extra virgin olive oil in all meals
Olive oil	0.4 [0.0–0.7]	
Virgin olive oil	0.5 [0.0–1.0]	
Extra virgin olive oil	1.2 [0.4–2.5]	
Sweets, pastries, sugars (cookies, cocoa, sugar, etc.)	2.0 [0.7–2.8]	Reduce and avoid consumption of processed foods with a high sugar content
Water (mL/day)	600.0 [500.0–600.0]	Water should be the beverage of choice in a healthy diet. Drink at least 1.5 L/day.
Natural fruits juices berverages (mL/day)	0.0 [0.0–28.0]	
Comercial fruits juices berverages (mL/day)	0.0 [0.0–0.0]	
Infusions (mL/day)	0.0 [0.0–200.0]	
Caffeinated drinks		It is recommended to reduce the consumption of caffeinated processed beverages
Coffee	1.1 [0.0–2.5]	
Tea	0.3 [0.0–0.1]	
Cola soft drinks	0.1 [0.0–0.0]	
Light cola soft drinks	0.2 [0.0–0.1]	
Alcoholic drinks	0.0 [0.0–0.1]	If alcohol is consumed, it should be in moderation

The results are expressed as the median with the 25th percentile and the 75th percentile in parentheses because data did not meet normality. The superscript * denotes those food groups for which AESAN recommends weekly consumption.

**Table 4:** Comparison of food group and drinks consumption based on migraine attack frequency and on disease-related disability according to the MIDAS Score in the studied population.

Food Groups	Migraine Attack Frequency				Disease-Related Disability According to the MIDAS Score				
	Infrequent *n* = 134	Frequent *n* = 49	Chronic *n* = 73	*p* Value	No Disability *n* = 16	Mild Disability *n* = 21	Moderate Disability *n* = 31	Severe Disability *n* = 192	*p* Value
Dairy products	2.0 [1.0–3.3]	2.0 [1.3–3.5]	1.7 [0.9–3.0]	0.203	2.0 [1.2–3.5]	2.4 [1.0–3.2]	2.4 [1.1–3.0]	2.0 [1.0–3.4]	0.872
Eggs	2.5 [2.5–6.3]	2.5 [1.0–2.5]	2.5 [1.0–7.0]	0.266	2.5 [2.5–7.0]	2.5 [2.5–4.0]	2.5 [1.0–3.3]	2.5 [1.0–4.8]	0.429
Meats	4.5 [3.0–7.0]	4.5 [3.0–7.0]	5.0 [3.5–7.0]	0.597	4.5 [2.4–7.6]	6.0 [3.5–7.0]	6.0 [2.8–7.3]	4.5 [3.0–7.0]	0.745
Fish	3.0 [2.0–7.0]	3.5 [2.0–7.0]	3.0 [2.0–7.0]	0.929	3.5 [2.4–7.3]	3.0 [2.0–4.5]	3.0 [1.0–7.0]	3.5 [2.0–7.0]	0.510
Vegetables	0.6 [0.1–1.1]	0.7 [0.1–0.9]	0.5 [0.0–1.1]	0.708	0.5 [0.3–1.1]	0.4 [0.0–0.9]	0.7 [0.2–1.2]	0.6 [0.0–1.1]	0.748
Fruits	1.6 [0.7–2.1]	1.1 [0.4–2.0]	1.1 [0.4–2.3]	0.251	1.4 [0.5–2.1]	1.4 [0.5–2.0]	1.1 [0.7–2.1]	1.4 [0.5–2.1]	0.950
Vegetables and fruits	2.1 [1.3–3.1]	1.8 [1.3–2.3]	2.0 [1.0–3.2]	0.215	2.0 [1.0–3.1]	2.0 [1.1–2.6]	2.0 [1.3–3.2]	2.0 [1.2–3.1]	0.833
Tubers	0.3 [0.04–0.5]	0.3 [0.1–0.6]	0.3 [0.0–0.7]	0.908	0.2 [0.1–0.4]	0.3 [0.0–0.7]	0.3 [0.1–1.0]	0.3 [0.0–0.6]	0.414
Legumes	2.0 [1.0–5.0]	2.0 [1.0–5.0]	2.5 [1.0–7.0]	0.772	2.0 [1.0–3.1]	2.0 [1.0–3.5]	2.0 [1.0–7.0]	2.5 [1.0–5.0]	0.918
Nuts	2.5 [0.0–7.0]	2.5 [1.0–7.0]	2.5 [0.0–7.0]	0.952	1.5 [0.0–5.0]	2.5 [2.0–7.0]	5.0 [1.0–7.5]	2.5 [0.0–7.0]	0.391
Cereals	1.9 [1.1–3.0]	1.3 [1.0–2.3]	1.7 [1.0–2.6]	0.258	1.7 [1.0–2.7]	1.6 [1.0–3.7]	2.0 [1.3–2.8]	1.6 [1.0–2.7]	0.724
Refined cereals	0.9 [0.3–1.5]	0.6 [0.1–1.1]	0.7 [0.3–1.3]	0.181	1.0 [0.6–2.0]	0.6 [0.3–1.3]	0.9 [0.3–1.9]	0.8 [0.3–1.3]	0.334
Whole grains	0.4 [0.0–1.0]	0.4 [0.0–1.0]	0.7 [0.0–1.1]	0.737	0.1 [0.0–0.6]	1.0 [0.0–1.1]	0.4 [0.0–1.0]	0.6 [0.0–1.0]	0.498
Oils	2.0 [1.0–2.7]	1.1 [1.0–2.5]	2.0 [1.0–3.0]	0.136	1.1 [1.0–2.1]	1.0 [1.0–2.5]	1.7 [1.0–3.3]	2.0 [1.0–2.7]	0.340
Olive oil	0.0 [0.0–0.6]	0.0 [0.0–0.1]	0.0 [0.0–1.0]	0.336	0.0 [0.0–0.6]	0.0 [0.0–0.0]	0.0 [0.0–1.0]	0.0 [0.0–1.0]	0.519
Virgin olive oil	0.0 [0.0–1.0]	0.0 [0.0–0.1]	0.0 [0.0–0.6]	0.052	0.1 [0.0–0.7]	0.0 [0.0–0.6]	0. 6 [0.0–1.0]	0.0 [0.0–1.0]	0.041 *
Extra virgin olive oil	1.0 [0.04–2.5]	1.0 [0.4–1.0]	1.0 [0.6–2.5]	0.376	1.0 [0.3–1.0]	1.0 [0.6–1.0]	1.0 [0.1–2.5]	1.0 [0.4–2.5]	0.746
Sweets, pastries, sugars	1.4 [0.7–2.9]	1.5 [0.6–2.6]	1.4 [1.0–2.5]	0.904	2.0 [0.9–3.1]	1.0 [0.1–2.0]	2.0 [0.8–3.5]	1.4 [0.8–2.5]	0.224
Water	600.0 [500.0–600.0]	600.0 [500.0–600.0]	600.0 [500.0–600.0]	0.864	500.0 [500.0–600.0]	600.0 [600.0–600.0]	600.0 [500.0–600.0]	600.0 [500.0–600.0]	0.223
Natural fruits juices beverages	0.0 [0.0–21.0]	0.0 [0.0–0.0]	0.0 [0.0–28.0]	0.674	0.0 [0.0–28.0]	0.0 [0.0–28.0]	0.0 [0.0–28.0]	0.0 [0.0–0.0]	0.711
Commercial fruits juices beverages	0.0 [0.0–0.0]	0.0 [0.0–0.0]	0.0 [0.0–0.0]	0.803	0.0 [0.0–7.0]	0.0 [0.0–0.0]	0.0 [0.0–0.0]	0.0 [0.0–0.0]	0.914
Infusions	14.0 [0.0–200.0]	0.0 [0.0–200.0]	0.0 [0.0–200.0]	0.626	0.0 [0.0–200.0]	0.0 [0.0–200.0]	0.0 [0.0–200.0]	0.0 [0.0–200.0]	0.810

The results are expressed as the median with the 25th percentile and the 75th percentile in parentheses as data did not meet normality. Comparison between groups showed no significant differences, except for virgin olive oil consumption; therefore, no post hoc analysis was carried out. Data were analyzed using Kruskal–Wallis test by ranks for multiple comparisons between groups. *p* values below 0.05 are highlighted with an asterisk (*).

**Table 5:** Comparison of foods and drinks associated as potential migraine episode triggers consumption based on migraine attack frequency in the studied population.

Food Groups	Migraine Attack Frequency				Disease-Related Disability According to the MIDAS Score				
	Infrequent *n* = 134	Frequent *n* = 49	Chronic *n* = 73	*p* Value	No Disability *n* = 16	Mild Disability *n* = 21	Moderate Disability *n* = 31	Severe Disability *n* = 192	*p* Value
Caffeine-rich drinks and foods									
Cups of coffee (60 mL)	1.0 [0.0–2.5]	1.0 [0.0–2.5]	0.6 [0.0–1.0]	0.064	1.0 [0.1–2.5]	1.0 [0.0–2.5]	0.6 [0.0–2.5]	1.0 [0.0–2.5]	0.539
Cups pf tea (220 mL)	0.0 [0.0–0.3]	0.0 [0.0–0.1]	0.0 [0.0–0.0]	0.214	0.0 [0.0–0.0]	0.0 [0.0–0.0]	0.0 [0.0–0.4]	0.0 [0.0–0.1]	0.126
Cola soft drinks (with caffein) (330 mL)	0.0 [0.0–0.0]	0.0 [0.0–0.0]	0.0 [0.0–0.0]	0.647	0.0 [0.0–0.0]	0.0 [0.0–0.1]	0.0 [0.0–0.0]	0.0 [0.0–0.0]	0.855
Light cola soft drinks (with caffein) (330 mL)	0.0 [0.0–0.0]	0.0 [0.0–0.1]	0.0 [0.0–0.0]	0.471	0.0 [0.0–0.2]	0.0 [0.0–0.0]	0.0 [0.0–0.1]	0.0 [0.0–0.04]	0.559
Chocolate (30 g)	0.1 [0.0–0.6]	0.1 [0.0–1.0]	0.0 [0.0–0.4]	0.032 *	0.0 [0.0–0.5]	0.0 [0.0–1.0]	0.0 [0.0–0.4]	0.1 [0.0–0.4]	0.988
Cocoa powder (50 g)	0.0 [0.0–0.1]	0.0 [0.0–0.0]	0.0 [0.0–0.1]	0.656	0.0 [0.0–0.2]	0.0 [0.0–0.0]	[0.0–0.4]	0.0 [0.0–0.0]	0.357
Total caffeine (mg/person)	130.0 [51.2–216.3]	115.0 [54.8–201.4]	80.0 [35.0–127.0]	0.046 *	98.1 [45.1–215.1]	200.0 [26.4–211.2]	80.0 [50.6–223.3]	90.0 [48.1–203.5]	0.974
Alcoholic drinks									
Alcoholic drinks: glass of red wine (100 mL); glass of white wine (100 mL); beer (330 mL); liqueurs, anise, or anisettes, etc. (150 mL); spirits: whisky, vodka, gin, cognac (50 mL)	0.0 [0.0–0.1]	0.0 [0.0–0.1]	0.0 [0.0–0.0]	0.016 *	0.1 [0.0–0.4]	0.0 [0.0–0.1]	0.0 [0.0–0.1]	0.0 [0.0–0.0]	0.042 *
Foods rich in biogenic amines or nitrites/nitrates									
Semi-cured cheeses (50 g)	0.0 [0.0–0.1]	0.0 [0.0–0.1]	0.0 [0.0–0.0]	0.008 **	0.0 [0.0–0.1]	0.0 [0.0–0.1]	0.0 [0.0–0.4]	0.0 [0.0–0.1]	0.353
Cured cheeses (50 g)	0.0 [0.0–0.1]	0.0 [0.0–0.4]	0.0 [ 0.0–0.0]	0.401	0.0 [0.0–0.4]	0.0 [0.0–0.4]	0.0 [0.0–0.5]	0.0 [0.0–0.0]	0.008 **
Cured meats (30 g)	0.1 [0.0–0.4]	0.1 [0.0–0.4]	0.0 [0.0–0.4]	0.151	0.1 [0.0–0.4]	0.1 [0.0–0.6]	0.1 [0.0–0.6]	0.0 [0.0–0.4]	0.020 *
Cooked ham (30 g)	0.4 [0.0–0.6]	0.1 [0.0–0.6]	0.1 [0.0–1.0]	0.553	0.4 [0.0–0.7]	0.4 [0.1–0.6]	0.4 [0.1–1.0]	0.1 [0.0–0.6]	0.046 *
Pickled foods (30 g)	0.0 [0.0–0.4]	0.1 [0.0–0.4]	0.0 [0.0–0.4]	0.212	0.0 [0.0–0.4]	0.0 [0.0- 0.4]	0.1 [0.0–1.0]	0.0 [0.0–0.4]	0.466

The results are expressed as the median with the 25th percentile and the 75th percentile in parentheses as data did not meet normality. The gram quantities of each food that constitute a standard serving are specified. Comparison between groups showed no significant differences. Data were analyzed using Kruskal–Wallis test by ranks for multiple comparisons between groups. *p* values under 0.05 are highlighted with one asterisk (*), while *p* values under 0.01 are highlighted with two asterisks (**).

## Data Availability

The original contributions presented in this study are included in the article/Supplementary Materials. Further inquiries can be directed to the corresponding author.

## Supplementary Materials

The following supporting information can be downloaded at: https://www.mdpi.com/article/10.3390/nu17040686/s1, Table S1. Comparison of food group consumption among consumers based on migraine attack frequency. Table S2. Comparison of foods and drinks associated as potential migraine episode triggers consumption among consumers based on migraine attack frequency. Table S3. Comparison of food group consumption among consumers based on disease-related disability according to the MIDAS scale. Table S4. Comparison of foods and drinks associated as potential migraine episode triggers consumption among consumers based on disease-related disability according to the MIDAS scale. Table S5. Average daily consumption frequency of different foods in the total sample. Table S6. Comparison of the total Dietary Diversity Score based on migraine attack frequency and disease-related disability according to the MIDAS scale. Figure S1. Caffeine consumption by migraine type and source.
