# Supplementary material for: An Assessment of the Dietary Habits of Individuals with Migraine Living in Spain: An Exploratory Observational Cross-Sectional Pilot Study
Source: Nutrients. 2025 Feb 14;17(4):686. doi: 10.3390/nu17040686 (PMC11857930; doi:10.3390/nu17040686)
Supplement: Supplementary file 1 [file nutrients-17-00686-s001.zip › nutrients-3410248-supplementary.pdf]

foods

MDPI

Article

Assessment of Dietary Habits in Individuals with Migraine Living in Spain: An Exploratory Observational Cross-Sectional Pilot Study

Supplementary tables

Table S1. Comparison of food group consumption among consumers based on migraine attack frequency.

Food groups	Total	Infrequent	Frequent	Chronic
Dairy products<br>C (n = 236; 90.8%)<br>NC: (n = 24; 9.2%)	2.3 [1.0 – 3.3]	2.3 [1.3 – 3.4]	2.2 [1.4 – 3.5]	2.3 [1.3 – 3.4]
Eggs<br>C: (n = 232; 89.2%)<br>NC: (n = 28; 10.8%)	3.6 [1.0 – 4.0]	2.5 [2.5 – 7.0]	2.5 [2.5 – 2.5]	2.5 [2.5 – 7.0]
Meats<br>C: (n = 234; 90%)<br>NC: (n = 26; 10%)	5.9 [3.0 – 7.0]	5.0 [3.5 – 7.0]	4.5 [3.0 – 7.0]	6.0 [3.5 – 7.5]
Fish<br>C: (n = 228; 87.7%)<br>NC: (n = 32; 12.3%)	4.8 [2.0 – 7.0]	3.5 [2.0 – 7.0]	4.0 [2.4 – 7.0]	4.0 [3.0 – 7.0]
Vegetables<br>C: (n = 190; 73.1%)<br>NC: (n = 70; 26.9%)	1.0 [0.0 – 1.1]	0.8 [0.4 – 1.3]	0.7 [0.5 – 1.1]	0.9 [0.5 – 1.3]
Fruits<br>C: (n = 240; 92.3%)<br>NC: (n = 20; 7.7%)	1.6 [0.5 – 2.1]	1.8 [0.8 – 2.1]	1.1 [0.6 – 2.0]	1.5 [1.0 – 2.3]
Vegetables and fruits<br>C: (n = 252; 97%)<br>NC: (n = 8; 3.0%)	2.6 [1.1 – 3.0]	2.1 [1.5 – 3.1]	1.9 [1.3 – 2.4]	2.0 [1.0 – 3.3]
Tubers<br>C: (n = 194; 74.6%)<br>NC: (n = 66; 25.4%)	0.5 [0.0 – 0.7]	0.4 [0.3 – 0.7]	0.3 [0.1 – 0.9]	0.6 [0.3 – 1.0]
Legumes<br>C: (n = 222; 85.4%)<br>NC: (n = 38; 14.6%)	3.9 [1.0 – 5.0]	2.5 [1.0 – 7.0]	2.5 [2.0 – 5.0]	3.5 [2.0 – 7.0]

Nuts<br>C: (n = 192; 73.8%)<br>NC: (n = 68; 26.2%)	4.7 [0.0 – 7.0]	5.0 [2.5 – 7.0]	3.5 [2.0 – 7.0]	5.0 [2.0 – 7.0]
Cereals<br>C: (n = 251; 96.5%)<br>NC: (n = 9; 3.5%)	2.1 [1.0 – 2.8]	2.0 [1.1 – 3.0]	1.3 [1.0 – 2.3]	1.9 [1.3 – 2.7]
Refined cereals<br>C: (n = 223; 85.8%)<br>NC: (n = 37; 14.2%)	1.0 [0.3 – 1.3]	1.0 [0.6 – 1.7]	0.8 [0.3 – 1.3]	0.9 [0.4 – 1.5]
Whole grains<br>C: (n = 166; 63.8%)<br>NC: (n = 94; 36.2%)	0.7 [0.0 – 1.0]	1.0 [0.6 – 1.3]	1.0 [0.4 – 1.1]	1.0 [0.7 – 1.3]
Oils<br>C: (n = 257; 98.8%)<br>NC: (n = 3; 1.2%)	2.3 [1.0 – 2.5]	2.0 [1.0 – 2.9]	1.1 [1.0 – 2.5]	2.0 [1.0 – 3.0]
Olive oil<br>C: (n = 88; 33.8%)<br>NC: (n = 172; 66.2%)	0.4 [0.0 – 0.7]	1.0 [0.6 – 2.5]	1.0 [0.6 – 1.0]	1.0 [1.0 – 2.5]
Virgin olive oil<br>C: (n = 103; 39.6%)<br>NC: (n = 157; 60.4%)	0.5 [0.0 – 1.0]	1.0 [1.0 – 2.5]	0.6 [0.1 – 1.0]	1.0 [0.6 – 2.2]
Extra virgin olive oil<br>C: (n = 209; 80.4%)<br>NC: (n = 51; 19.6%)	1.2 [0.4 – 2.5]	1.0 [1.0 – 2.5]	1.0 [1.0 – 2.1]	1.0 [1.0 – 2.5]
Sunflower oil<br>C: (n = 33; 12.7%)<br>NC: (n = 227; 87.3%)	0.0 [0.0 – 0.0]	1.0 [0.1 – 1.0]	0.1 [0.1 – 0.4]	0.4 [0.4 – 1.0]
High oleic sunflower oil<br>C: (n = 16; 6.2%)<br>NC: (n = 244; 93.8%)	0.0 [0.0 – 0.0]	1.0 [0.1 – 1.0]	0.1 [0.1 – 0.1]	1.0 [0.4 – 1.0]
Coconut oil<br>C: (n = 15; 5.8%)<br>NC: (n = 245; 94.2%)	0.0 [0.0 – 0.0]	0.1 [0.1 – 1.0]	0.1 [0.1 – 0.1]	1.0 [1.0 – 1.0]
Sweets, pastries, sugars<br>C: (n = 231; 88.8%)<br>NC: (n = 29; 11.2%)	2.0 [0.7 – 2.8]	2.0 [1.0 – 3.0]	2.0 [1.0 – 3.1]	1.6 [1.0 – 3.0]

The results are expressed as the median with the 25th percentile and the 75th percentile in parentheses because data did not meet normality. For each food group, the total number of consumers (*n*) is provided, with the percentage of non-consumers in parentheses. C: consumers; NC: non-consumers.

Table S2. Comparison of foods and drinks associated as potential migraine episode triggers consumption among consumers based on migraine attack frequency.

Food groups	Total	Infrequent	Frequent	Chronic
**Caffeine-rich drinks and foods**				
Cups of coffee<br>C: (n = 177; 68.1%)<br>NC: (n = 83; 31.9%)	1.1 [0.0 – 2.5]	2.5 [1.0 – 2.5]	1.0 [1.0 – 2.5]	1.0 [0.9 – 1.4]
Cups of tea<br>C: (n = 73; 28.1%)<br>NC: (n = 187; 71.9%)	0.3 [0.0 – 0.1]	1.0 [0.4 – 1.0]	1.0 [0.2 – 1.0]	0.4 [0.1 – 1.0]
Cola soft drinks (with caffein)<br>C: (n = 55; 21.2%)<br>NC: (n = 205; 78.8%)	0.1 [0.0 – 0.0]	[0.1 – 0.5]	0.3 [0.1 – 1.0]	0.6 [0.1 – 1.0]
Light cola soft drinks (with caffein)<br>C: (n = 68; 26.2%)<br>NC: (n = 192; 73.8%)	0.2 [0.0 – 0.1]	0.4 [0.1 – 0.4]	[0.1 – 1.0]	0.4 [0.1 – 1.0]
Chocolate<br>C: (n = 133; 51.2%)<br>NC: (n = 127; 48.8%)	0.1 [0.0 – 0.4]	0.4 [0.1 – 1.0]	0.4 [0.1 – 1.0]	0.4 [0.1 – 1.0]
Cocoa powder<br>C: (n = 68; 26.2%)<br>NC: (n = 192; 73.8%)	0.0 [0.0 – 0.1]	0.8 [0.4 – 1.0]	0.4 [0.1 – 0.7]	1.0 [0.4 – 1.0]
**Alcoholic drinks**				
Alcoholic drinks<br>C: (n = 66; 25.4%)<br>NC: (n = 194; 74.6%)	0.0 [0.0 – 0.1]	0.1 [0.1 – 0.4]	0.1 [0.1 – 0.4]	0.1 [0.1 – 0.4]
**Foods rich in biogenic amines or nitrites/nitrates**				
Semi-cured cheeses<br>C: (n = 86; 33.1%)<br>NC: (n = 174; 66.9%)	0.1 [0.0 – 0.4]	0.4 [0.1 – 0.8]	1.0 [0.1 – 1.0]	0.1 [0.1 – 0.4]
Cured cheeses<br>C: (n = 76; 29.2%)<br>NC: (n = 184; 70.8%)	0.0 [0.0 – 0.1]	0.4 [0.1 – 1.0]	1.0 [0.4 – 1.8]	0.1 [0.1 – 0.4]
Cured meats<br>C: (n = 129; 49.6%)<br>NC: (n = 131; 50.4%)	0.1 [0.0 – 0.4]	0.4 [0.1 – 1.0]	0.4 [0.1 – 0.8]	0.4 [0.1 – 1.0]
Cooked ham<br>C: (n = 175; 67.3%)<br>NC: (n = 85; 32.7%)	0.0 [0.0 – 0.1]	0.4 [0.4 – 1.0]	0.4 [0.1 – 1.0]	0.5 [0.1 – 1.0]

Pickled foods<br>C: (n = 126; 48.5%)<br>NC: (n = 134; 51.5%)	0.1 [0.0 – 0.4]	0.4 [0.1 – 1.0]	0.4 [0.1 – 1.0]	0.4 [0.1 – 1.0]

The results are expressed as the median with the 25th percentile and the 75th percentile in parentheses because data did not meet normality. For each food group, the total number of consumers (*n*) is provided, with the percentage of non-consumers in parentheses. C: consumers; NC: non-consumers.

Table S3. Comparison of food group consumption among consumers based on disease-related disability according to the MIDAS scale.

Food groups	Total	No disability *n* = 16	Mild disability *n* = 21	Moderate disability *n* = 31	Severe disability *n* = 192
Dairy products<br>C (n = 236; 90.8%)<br>NC: (n = 24; 9.2%)	2.3 [1.0 – 3.3]	2.0 [1.2 – 3.5]	2.5 [1.6 – 3.2]	2.5 [1.7 – 3.0]	2.1 [1.0 – 3.5]
Eggs<br>C: (n = 232; 89.2%)<br>NC: (n = 28; 10.8%)	3.6 [1.0 – 4.0]	2.5 [2.5 – 7.0]	2.5 [2.5 – 6.3]	2.5 [2.5 – 4.0]	2.5 [2.5 – 7.0]
Meats<br>C: (n = 234; 90%)<br>NC: (n = 26; 10%)	5.9 [3.0 – 7.0]	4.5 [3.5 – 8.0]	6.0 [4.3 – 7.0]	6.3 [3.0 – 7.5]	5.0 [3.5 – 7.0]
Fish<br>C: (n = 228; 87.7%)<br>NC: (n = 32; 12.3%)	4.8 [2.0 – 7.0]	3.5 [2.8 – 7.5]	3.5 [2.5 – 4.5]	3.0 [2.0 – 7.5]	4.0 [2.0 – 7.0]
Vegetables<br>C: (n = 190; 73.1%)<br>NC: (n = 70; 26.9%)	1.0 [0.0 – 1.1]	0.7 [0.5 – 1.3]	0.7 [0.4 – 1.1]	0.9 [0.5 – 1.3]	0.8 [0.5 – 1.3]
Fruits<br>C: (n = 240; 92.3%)<br>NC: (n = 20; 7.7%)	1.6 [0.5 – 2.1]	1.4 [0.5 – 2.1]	1.4 [0.8 – 2.0]	1.2 [0.8 – 2.2]	1.5 [0.8 – 2.3]
Vegetables and fruits<br>C: (n = 252; 97%)<br>NC: (n = 8; 3.0%)	2.6 [1.1 – 3.0]	2.0 [1.0 – 3.1]	2.0 [1.1 – 2.6]	2.0 [1.3 – 3.3]	2.1 [1.3 – 3.1]
Tubers<br>C: (n = 194; 74.6%)<br>NC: (n = 66; 25.4%)	0.5 [0.0 – 0.7]	0.3 [0.1 – 0.4]	0.4 [0.3 – 0.7]	0.5 [0.3 – 1.0]	0.4 [0.2 – 0.7]
Legumes<br>C: (n = 222; 85.4%)<br>NC: (n = 38; 14.6%)	3.9 [1.0 – 5.0]	2.0 [1.3 – 4.4]	2.5 [1.0 – 4.3]	4.5 [1.0 – 7.0]	2.5 [2.0 – 7.0]
Nuts<br>C: (n = 192; 73.8%)<br>NC: (n = 68; 26.2%)	4.7 [0.0 – 7.0]	5.0 [2.5 – 7.0]	2.5 [2.5 – 7.0]	7.0 [2.4 – 9.5]	4.0 [2.0 – 7.0]
Cereals<br>C: (n = 251; 96.5%)<br>NC: (n = 9; 3.5%)	2.1 [1.0 – 2.8]	1.7 [1.0 – 2.7]	2.0 [1.0 – 3.8]	2.0 [1.3 – 2.8]	1.7 [1.0 – 2.7]

Refined cereals<br>C: (n = 223; 85.8%)<br>NC: (n = 37; 14.2%)	1.0 [0.3 – 1.3]	1.0 [0.6 – 2.0]	1.3 [0.5 – 1.7]	1.3 [0.5 – 2.0]	1.0 [0.4 – 1.4]
Whole grains<br>C: (n = 166; 63.8%)<br>NC: (n = 94; 36.2%)	0.7 [0.0 – 1.0]	0.5 [0.2 – 0.9]	1.0 [1.0 – 2.3]	1.0 [0.7 – 1.2]	1.0 [0.6 – 1.3]
Oils<br>C: (n = 257; 98.8%)<br>NC: (n = 3; 1.2%)	2.3 [1.0 – 2.5]	1.1 [1.0 – 2.1]	1.1 [1.0 – 2.5]	1.7 [1.0 – 3.3]	2.0 [1.0 – 2.7]
Olive oil<br>C: (n = 88; 33.8%)<br>NC: (n = 172; 66.2%)	0.4 [0.0 – 0.7]	0.8 [0.6 – 1.0]	1.0 [0.6 – 1.0]	1.0 [0.7 – 1.0]	1.0 [1.0 – 2.5]
Virgin olive oil<br>C: (n = 103; 39.6%)<br>NC: (n = 157; 60.4%)	0.5 [0.0 – 1.0]	0.8 [0.5 – 1.0]	0.8 [0.6 – 1.0]	1.0 [0.6 – 2.5]	1.0 [0.6 – 2.5]
Extra virgin olive oil<br>C: (n = 209; 80.4%)<br>NC: (n = 51; 19.6%)	1.2 [0.4 – 2.5]	1.0 [0.6 – 1.0]	1.0 [1.0 – 2.5]	1.0 [0.6 – 2.5]	1.0 [1.0 – 2.5]
Sunflower oil<br>C: (n = 33; 12.7%)<br>NC: (n = 227; 87.3%)	0.0 [0.0 – 0.0]	1.0 [1.0 – 1.0]	3.0 [3.0 – 3.0]	0.1 [0.1 – 0.1]	0.6 [0.2 – 1.0]
High oleic sunflower oil<br>C: (n = 16; 6.2%)<br>NC: (n = 244; 93.8%)	0.0 [0.0 – 0.0]	-	-	0.1 [0.1 – 0.6]	1.0 [0.4 – 1.0]
Coconut oil<br>C: (n = 15; 5.8%)<br>NC: (n = 245; 94.2%)	0.0 [0.0 – 0.0]	-	-	0.1 [0.1 – 0.6]	1.0 [0.1 – 1.0]
Sweets, pastries, sugars<br>C: (n = 231; 88.8%)<br>NC: (n = 29; 11.2%)	2.0 [0.7 – 2.8]	2.1 [1.6 – 3.3]	1.3 [1.0 – 2.4]	2.4 [1.2 – 3.9]	1.5 [1.0 – 3.0]

The results are expressed as the median with the 25th percentile and the 75th percentile in parentheses as data did not meet normality. For each food group, the total number of consumers (n) is provided, with the percentage of non-consumers in parentheses. Empty cells indicate that there were no consumers for this specific food item. C: consumers; NC: non-consumers.

Table S4. Comparison of foods and drinks associated as potential migraine episode triggers consumption among consumers based on disease-related disability according to the MIDAS scale.

Food groups	Total	No disability *n* = 16	Mild disability *n* = 21	Moderate disability *n* = 31	Severe disability *n* = 192
**Caffeine-rich drinks and foods**					
Cups of coffee<br>C: (n = 177; 68.1%)<br>NC: (n = 83; 31.9%)	1.1 [0.0 – 2.5]	1.0 [1.0 – 2.5]	2.5 [2.5 – 2.5]	1.8 [1.0 – 2.5]	1.0 [1.0 – 2.5]
Cups of tea<br>C: (n = 73; 28.1%)<br>NC: (n = 187; 71.9%)	0.3 [0.0 – 0.1]	0.1 [0.1 – 0.1]	0.8 [0.5 – 1.4]	1.0 [0.5 – 1.0]	1.0 [0.1 – 1.0]
Cola soft drinks (with caffein)<br>C: (n = 55; 21.2%)<br>NC: (n = 205; 78.8%)	0.1 [0.0 – 0.0]	0.1 [0.1 – 0.1]	0.1 [0.1 – 0.3]	0.3 [0.1 – 0.4]	0.4 [0.1 – 1.0]
Light cola soft drinks (with caffein)<br>C: (n = 68; 26.2%)<br>NC: (n = 192; 73.8%)	0.2 [0.0 – 0.1]	0.7 [0.2 – 1.0]	0.1 [0.1 – 0.1]	1.0 [0.1 – 1.0]	0.4 [0.1 – 1.0]
Chocolate<br>C: (n = 133; 51.2%)<br>NC: (n = 127; 48.8%)	0.1 [0.0 – 0.4]	1.0 [0.3 – 1.0]	1.0 [0.2 – 1.0]	0.4 [0.3 – 1.0]	0.4 [0.1 – 1.0]
Cocoa powder<br>C: (n = 68; 26.2%)<br>NC: (n = 192; 73.8%)	0.0 [0.0 – 0.1]	0.4 [0.4 – 1.0]	0.4 [0.1 – 1.0]	1.0 [0.3 – 1.0]	1.0 [0.2 – 1.0]
**Alcoholic drinks**					
Alcoholic drinks<br>C: (n = 66; 25.4%)<br>NC: (n = 194; 74.6%)	0.0 [0.0 – 0.1]	0.4 [0.3 – 0.5]	0.4 [0.2 – 0.4]	0.1 [0.1 – 0.4]	0.1 [0.1 – 0.4]
**Foods rich in biogenic amines or nitrites/nitrates**					
Semi-cured cheeses<br>C: (n = 86; 33.1%)<br>NC: (n = 174; 66.9%)	0.1 [0.0 – 0.4]	0.1 [0.1 – 0.8]	0.4 [0.1 – 0.6]	0.4 [0.2 – 1.0]	0.4 [0.1 – 1.0]
Cured cheeses<br>C: (n = 76; 29.2%)<br>NC: (n = 184; 70.8%)	0.0 [0.0 – 0.1]	1.0 [0.4 – 1.0]	0.6 [0.1 – 1.0]	0.6 [0.3 – 1.0]	0.4 [0.1 – 1.0]
Cured meats<br>C: (n = 129; 49.6%)<br>NC: (n = 131; 50.4%)	0.1 [0.0 – 0.4]	0.4 [0.1 – 0.4]	0.4 [0.1 – 0.8]	0.4 [0.1 – 1.0]	0.4 [0.1 – 1.0]

Cooked ham<br>C: (n = 175; 67.3%)<br>NC: (n = 85; 32.7%)	0.0 [0.0 – 0.1]	0.6 [0.4 – 1.0]	0.4 [0.4 – 0.9]	0.6 [0.4 – 1.0]	0.4 [0.1 – 1.0]
Pickled foods<br>C: (n = 126; 48.5%)<br>NC: (n = 134; 51.5%)	0.1 [0.0 – 0.4]	0.4 [0.3 – 0.8]	0.4 [0.1 – 0.4]	0.7 [0.2 – 1.0]	0.4 [0.1 – 0.4]

The results are expressed as the median with the 25th percentile and the 75th percentile in parentheses because data did not meet normality. For each food group, the total number of consumers (n) is provided, with the percentage of non-consumers in parentheses. C: consumers; NC: non-consumers.

Table S5. Average daily consumption frequency of different foods in the total sample.

Foods	Total
**Caffeine-rich drinks and foods**	
Cups of coffee	1.0 [0.0 – 2.5]
Cups of tea	0.0 [0.0 – 0.1]
Cola soft drinks (with caffein)	0.0 [0.0 – 0.0]
Light cola soft drinks (with caffein)	0.0 [0.0 – 0.1]
Chocolate	0.1 [0.0 – 0.4]
Cocoa powder	0.0 [0.0 – 0.1]
**Alcoholic drinks**	
Alcoholic drinks	0.0 [0.0 – 0.1]
**Foods rich in biogenic amines or nitrites/nitrates**	
Semi-cured cheeses	0.0 [0.0 – 0.1]
Cured cheeses	0.0 [0.0 – 0.1]
Cured meats	0.0 [0.0 – 0.4]
Cooked ham	0.4 [0.0 – 0.6]
Pickled foods	0.0 [0.0 – 0.4]

The results are expressed as the median with the 25th percentile and the 75th percentile in parentheses because as data did not meet normality. The gram quantities of each food that constitute a standard serving are specified.

Table S6. Comparison of the total Dietary Diversity Score based on migraine attack frequency and disease-related disability according to the MIDAS scale.

	Total	Type of migraine				p value (between groups)
**Dietary Diversity Score (DDS)**	10.0 [8.0 – 11.0]	**Infrequent**	**Frequent**	**Chronic**		
		10.0 [9.0 – 11.0]	10.0 [8.0 – 11.0]	10.0 [8.0 – 11.0]		0.830
		**MIDAS Scale**				
		**No disability**	**Mild disability**	**Moderate disability**	**Severe disability**	
		10.5 [8.0 – 11.0]	10.0 [9.0 – 11.0]	10.0 [9.0 – 11.0]	10.0 [8.0 – 11.0]	0.965

The results are expressed as the median with the 25th percentile and the 75th percentile in parentheses because data did not meet normality. Comparison between groups showed no significant differences. No *post hoc* analysis was carried out. Data were analysed using Kruskal–Wallis test by ranks for multiple comparisons between groups.

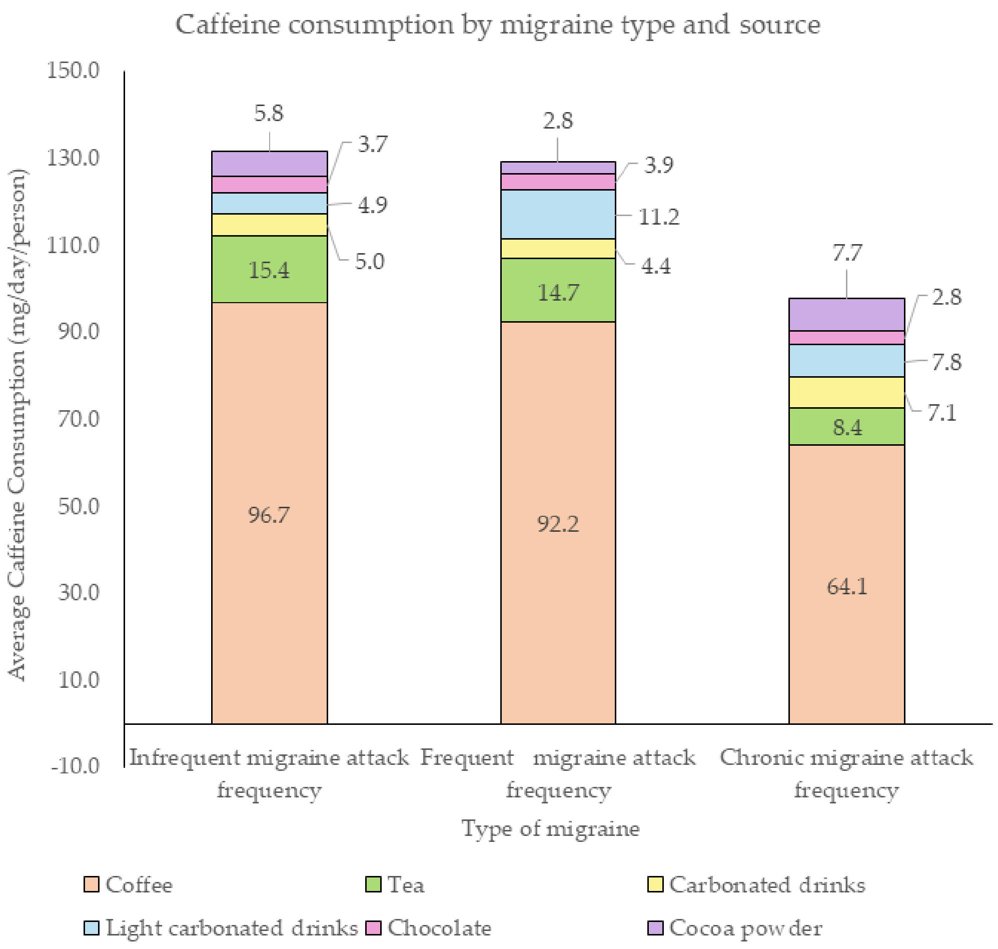

Figure S1. Caffeine consumption by migraine type and source
